# Unraveling fine-scale habitat use for secretive species: When and where toads are found when not breeding

**DOI:** 10.1371/journal.pone.0205304

**Published:** 2018-10-08

**Authors:** Karoline C. Gilioli, Marc Kéry, Murilo Guimarães

**Affiliations:** 1 Departamento de Zoologia, Universidade Federal do Rio Grande do Sul, Porto Alegre, RS, Brazil; 2 Swiss Ornithological Institute, Sempach, Switzerland; Universitat Trier, GERMANY

## Abstract

A good understanding of species-habitat associations, or habitat use, is required to establish conservation strategies for any species. Many amphibian species are elusive and most information concerning amphibian habitat use comes from breeding sites where they are comparatively easy to find and study. Knowledge about retreat sites is extremely limited for most species and for the greater part of the year. For such species, it is especially important to factor in detection probability in habitat analyses, because otherwise distorted views about habitat preferences may result, e.g., when a species is more visible in habitat type B than in A, even though A may be preferred. The South American red-belly toad, *Melanophryniscus pachyrhynus*, is a range-restricted species from Southern Brazil and Uruguay that inhabits open areas with rocky outcrops and is usually seen only during explosive breeding events. Here we studied the fine-scale habitat use of the red-belly toad outside of the breeding season to identify retreat sites and test for the importance of accounting for species imperfect detection, using Bayesian occupancy models. We identified shrub density and the number of loose rocks as important predictors of occupancy, while detection probability was highest at intermediate temperatures. Considering the harsh (dry and hot) conditions of rocky outcrops, shrubs and loose rocks may both work as important refuges, besides providing food resources and protecting against predation. Rocky outcrops have been suffering changes in habitat configuration and we identify nonbreeding habitat preferences at a fine scale, which may help to promote population persistence, and highlight the importance of accounting for imperfect detection when studying secretive species.

## Introduction

Identifying the main factors predicting a species' distribution has been widely applied to wildlife management and recognized as being critical for guiding conservation efforts [1–3]. At the same time, a clear understanding about the spatial scale at which the habitat is important for a species is needed [4]. Broad-scale (e.g., global) studies usually focus on very broad patterns of species occurrence and have revealed fast range declines of an increasing number of species [5]. However, the observed broad-scale distribution of a species may be poorly estimated without accounting for fine-scale variation of occurrence within the broad distribution limits of a species [6].

Fine-scale variation of occurrence is governed by the effects relevant to individuals [7] and includes different factors, such as local resource use and microclimate [8]. Such studies provide details about biological mechanisms underlying species distributions [9] and are critical to habitat management solutions that may not be afforded by broader scale approaches [10–11]. A process occurring at the scale of a local population may determine species decline and recovery [12], since the habitat matrix occupied by a species at broad scales depends on the quality of the local habitat [13]. Moreover, it is at the local scale where conservation measures can act, not usually at the scale of the entire distribution of a species [12]. Therefore, a clear understanding of fine-scale habitat use of a study species is important both for scientific and for conservation purposes.

Amphibians are among the most endangered vertebrate groups and losses of amphibian populations have been a global concern for decades [14–15], where habitat loss is arguably the major threat to this group. Most previous studies have not provided clear insights about how habitat loss affects amphibian local populations in a mechanistic way [16], hence, local studies are particularly important to reveal key aspects for population persistence [17–18]. In addition, the relatively low dispersal ability of amphibians makes fine-scale habitat studies particularly important.

In amphibians, there is a huge bias in habitat studies from reproductive sites during the calling and breeding season [19]. The reason for this is that amphibian habitat use is comparatively easy to study on breeding sites where species density is high and they are easier to find, for instance [20–22]. However, most species spend by far the largest part of the year in entirely different habitats where they are much more difficult to study. This is because they do neither congregate in large numbers nor call, and often remain hidden in refuges to retain humidity and to avoid predators [23]. Thus, a habitat may be erroneously taken to be preferred, when in fact a species is simply more detectable there. Seasonal patterns in behavior also largely influence amphibian detection in the field [18, 24] and these characteristics altogether highlight the importance of explicitly accounting for spatial and temporal variation in the probability of detection of a species [25].

In the context of species distribution, detection probability is usually taken to be the probability of detecting at least one individual of the species during a particular sampling occasion at a site where it is present [26]. Models relying on detection/non-detection data are able to account for bias caused when failing to observe the target species at occupied sites, i.e., for false-negative errors. Authors have emphasized the importance of accounting for detection probability to strengthen the inferences in herpetofaunal studies [27–28], particularly those dealing with elusive species, which most herpetofauna belongs to.

The South American red-belly toads (*Melanophryniscus* spp.) are secretive neotropical endemic anurans in some cases restricted to just a handful of known sites in subtropical and tropical South America [29]. Twenty-nine species of red-belly toads are recognized [30], and almost all information available is restricted to temporary aquatic environments that are created after intense rainfall, where explosive breeding occurs for short time periods (see [31] and references therein). Information on habitat use during the non-breeding period is still lacking for most species. *Melanophryniscus pachyrhynus* is associated with rocky outcrop environments in upland open areas in the South American Pampa biome [32–33], in the southernmost part of Brazil and Uruguay. Much of its ecology remains unknown and the species is listed as Data Deficient in the IUCN Red List [34]. Information about habitat preferences outside breeding sites is limited to observations of specimens found under rocks near small watercourses [32].

Rocky outcrops form a specialized suite of habitats (e.g. rock pools, loose rocks, rock crevices) that connects with gallery forests, grasslands, and their vegetative succession stages [35]. They represent extreme habitats demanding adaptations to survive high thermal amplitudes, heavy winds, and drought [36]. These rock elements comprise high-biodiversity habitats with many endemic species [37–38] that have been suffering from human activities, including livestock, fire, and forestry [39]. Given the uniqueness of those habitats and the elusive habits of the genus *Melanophryniscus* [29], there is thus an urgent need to fill information gaps in the spatial distribution and ecological requirements of these toads [40].

Considering the lack of knowledge about amphibian retreat sites, we assess habitat use for the red-belly toad *Melanophryniscus pachyrhynus* focusing on the non-breeding period. Our goal is two-fold, where first (i) we investigate which characteristics of the retreat sites—rocky outcrops—enable the red-belly toad persistence and then (ii) we test the importance of accounting for false negative errors when determining species occupancy probability.

## Materials and methods

### Study area and sampling design

**Fig 1:**
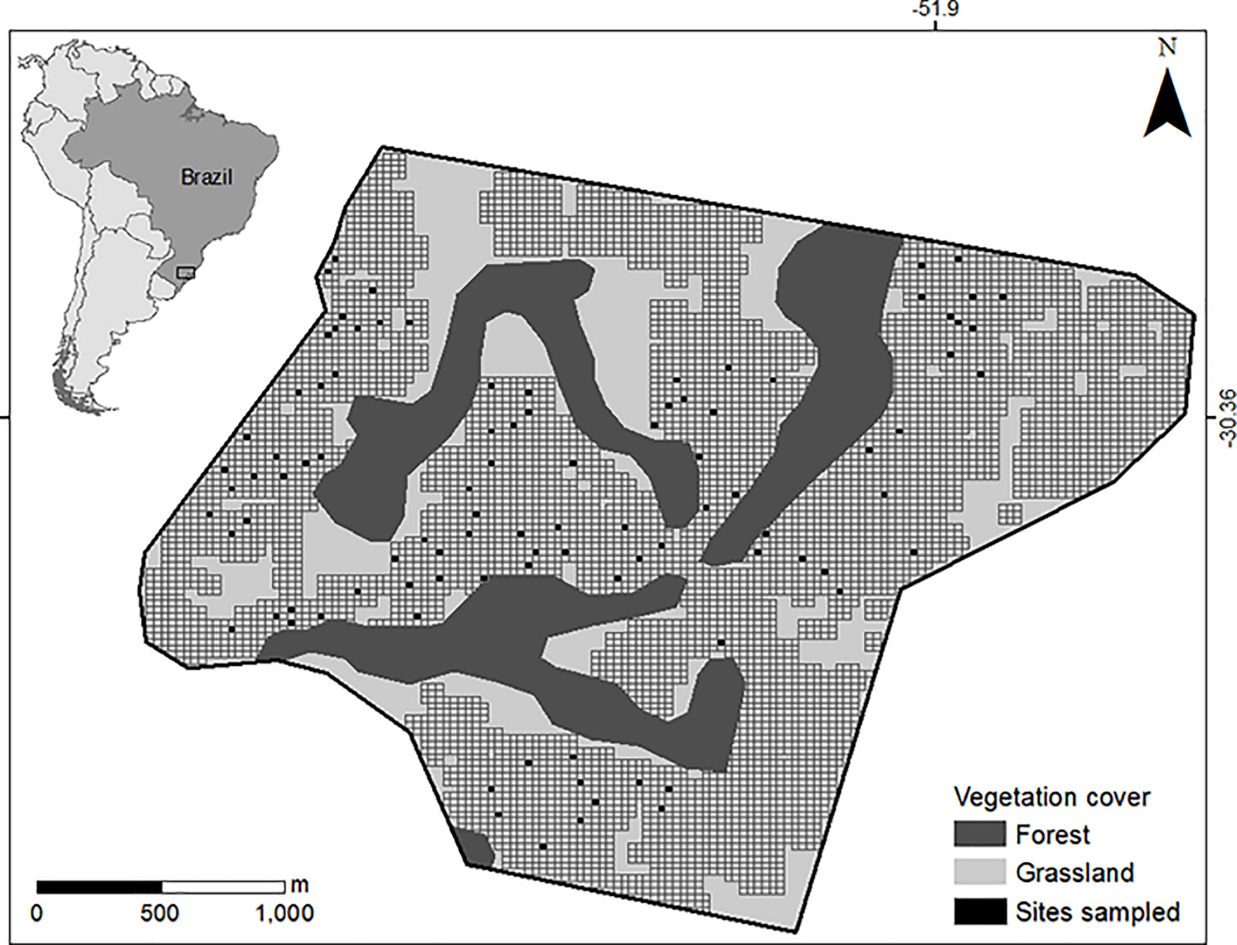
Study area showing the 30x30 meter grid cells and the 96 surveyed sites (cells in black). Grassland and Forest landscape features discerned based on [42].

Our study was conducted in the South American Pampa biome, in the state of Rio Grande do Sul, southern Brazil (Fig 1).The climate is subtropical temperate with well-defined seasons, ranging from dry and hot in the austral summer (December–February) to cold and humid in the winter (June–August). High annual and daily thermal amplitudes are observed. Temperatures can vary from 4 °C to 28 °C during a single day in winter, and reach 40 °C in the summer. Regular rainfall, totaling between 1,200 and 1,600 mm annually, occurs throughout the year [41]. Our study area comprised a 500-ha area in a transition zone between Savanna and Seasonal Semi-deciduous Forest, at approx. 600 meters a.s.l. The area includes occasional slopes with hills and rocky outcrops surrounded by ephemeral ponds, grasslands, shrub vegetation and gallery forests (S1 Fig).

To study fine-scale toad occurrence we superimposed a 30x30 meters-grid composed of approximately 6,000 grid cells over a 30-meter resolution satellite image. We randomly selected 96 sites that were surveyed between one and five times each. Since the dispersive capacity of red-belly toads is still unknown, grid cell size, corresponding to our sampling sites, was defined based on similar studies with small amphibians [43]. We excluded forested cells and also those strongly altered by anthropogenic activities, since our goal was to determine the fine-scale species’ preferences in natural open areas with rocky outcrops, corresponding to previous knowledge about the biology of the species [31]. Surveys took place from April to October 2016 for four days per month. We visited approximately 50 sites in each month, randomly chosen out the 96 sites. We used active visual searching [44] during the day, from 8 am to 6 pm, targeting adult individuals (Animal Welfare permit numbers: Chico Mendes Institute for Biodiversity Conservation license number 10341–1, Universidade Federal do Rio Grande do Sul Committee license number 30875) outside of the breeding period, thus avoiding biases from births and migration movements. Four trained observers (for a total of 10) were deployed monthly to search the species, mainly under rocks, the supposed microhabitat shelter for the species.

### Spatial and temporal covariates of toad occupancy and detection probability

As spatial covariates we considered the number of loose rocks (*rocks*), grass and bush density cover at 50 cm high (*vegetation*), bare rocky surface (*barerock*), and slope of the site (*siteslope*). The number of loose rocks we could lift on each site was counted and then averaged across multiple visits to the same site. We systematically selected 12 1.5x1.5-meter plots inside of each 900-m^2^ site to measure vegetation density and bare rocky surface by adapting the method of [45], see S2 Fig. To express fine-scale slope, we took the highest and lowest altitudes of the site using a GPS.

All spatial covariates, except site slope, were also used to account for variation in detection probability in our model. Besides that, we included air temperature (*temperature*) and its quadratic term (*temperature*^*2*^), air humidity (*humidity*), and rainfall (*rainfall*). Additionally, we recorded sampling hour (*hour*, expressed as minutes after midnight) and sampling effort (*samplingeffort*) because the activity of our study species and sampling effort could both influence the probability of detecting the species. Finally, we included a random term (*eps*.*p*) assumed to be normally distributed with hyperparameters mean 0 and variance *tau*.*alpha0*. The random effect term addresses potential unmodeled heterogeneity among sites by covariates since it accounts for uncertainty and reduces bias when it is not possible to know or measure all sources of variation [46–47].

### Statistical analysis

We fitted a single-season occupancy model [26] to jointly estimate species occupancy and detection probability. Occupancy models formally distinguish between two processes in the model structure, the ecological submodel for the presence or absence of the species and the observational submodel for measurement or observation process, represented by one or more parameters for imperfect detection [48]. The presence/absence of the species at site *i* is modeled as binary latent occurrence indicator (*z*_*i*_*)* treated as a Bernoulli random variable governed by parameter *ψ* (occupancy probability).

The measurement process is expressed by treating the observed detection or non-detection at site *i* during survey *j* as another Bernoulli random variable (*y*_*i*,*j*_*)* with a success probability that is the product of the species occurrence indicator (*z*_*i*_) and detection probability *p*_*i*,*j*_ at site *i* during survey *j*. The two parameters (*ψ*_*i*_ and *p*_*i*,*j*_) are separately estimable through replicate visits. Effects of covariates were expressed via a *logit*-link function for site-specific covariates to model occupancy probability and for site- and time-specific covariates to explain variability in detection probability [26]. All covariates were scaled to have zero mean and unit variance. We considered only the main effects of these covariates and all covariate pairs presented correlation below 0.7. Our model for occupancy (1) at each site *i*, and for detection (2) at each site *i* and visit *j* were respectively:

$$logit(\psi_{i})=\beta_{0}+\beta_{1}*\;{rocks}_{i}+\beta_{2}*{barerock}_{i}+\beta_{3}*{vegetation}_{i}+\beta_{4}\;{siteslope}_{i}$$ (1)

$$\begin{array}{l} logit\left(p_{i,j}\right)=\alpha_{0}+\alpha_{1}*humidity_{i,j}+\alpha_{2}*temperature_{i,j}+\alpha_{3}*temperature^{2}{}_{i,j}+\\ \alpha_{4}*rocks_{i}+\alpha_{5}*barerock_{i}+\alpha_{6}*vegetation_{i}+\alpha_{7}*samplingeffort_{i,j}+\\ \;\alpha_{8}*hour_{i,j}+\alpha_{9}*rainfall_{i,j}+eps.p_{i} \end{array}$$ (2)

We used a Bayesian mode of inference for the parameters in the model with Markov Chain Monte Carlo (MCMC) [48]. We ran three chains of 200,000 iterations each, with a 100,000 iterations as a burnin period and a thinning rate of 50, which resulted in 6,000 samples from the posterior distribution of each parameter. We also estimated the following derived quantities: the finite-sample number of occupied sites among the 96 surveyed sites (N.occu), which is the sum of the latent variable (*z*) over the 96 study sites, the finite-sample proportion of occupied sites among sampling surveys (ψ^fs^) expressed as N.occu divided by the number of sites, the average occupancy (psi.mean) and detection probability (p.mean), and the probability of detecting the species at least once in *n* surveys (p*), given its presence (see [49]). The latter is given by 1−(1−p)^*n*^, where *p* is the average detection probability (mean.p) per survey and where we varied survey number (*n)* from 1 to 5, corresponding to the minimum and maximum number of surveys in our study.

To gauge the effects of ignoring imperfect detection in a species distribution model (SDM), we also fitted a comparable, traditional SDM without a submodel for detection, i.e., a logistic regression of the observed occupancy status of each site on all covariates in our study. For temporal covariates (humidity, temperature, sampling effort, hour and rainfall), we took the mean over repeated visits.

We implemented models using the BUGS language [50] in JAGS [51], which we ran from R (R Core Team 2016) through the jagsUI package [52]. We adopted vague priors for all parameters (see JAGS code in S3 Fig). We determined whether chains had converged by visually examining trace plots and by the Brooks–Gelman–Rubin statistic [53], which was < 1.1 for all parameters. We present posterior means, standard deviations and 95% credible intervals.

## Results

### Data overview

On average, we surveyed sites three times and we detected the red-belly toad in 38 out of the 96 sites, which represents an observed proportion of occupied sites of 40%. Almost all individuals (95% of n = 65) were found under loose rocks in contact with moist soil, among the vegetation on the rocky outcrop edges. Occasionally, we observed two or more individuals under the same rock or sharing the same refuge with other species of invertebrates (e.g. spiders and ants), amphibians (e.g. *Scinax fuscovarius*), and reptiles, including lizards (e.g. *Contomastix lacertoides* and *Cercosaura schreibersii*) and snakes (e.g. *Sibynomorphus ventrimaculatus*).

### Habitat relationships in the red-belly toad

**Fig 2:**
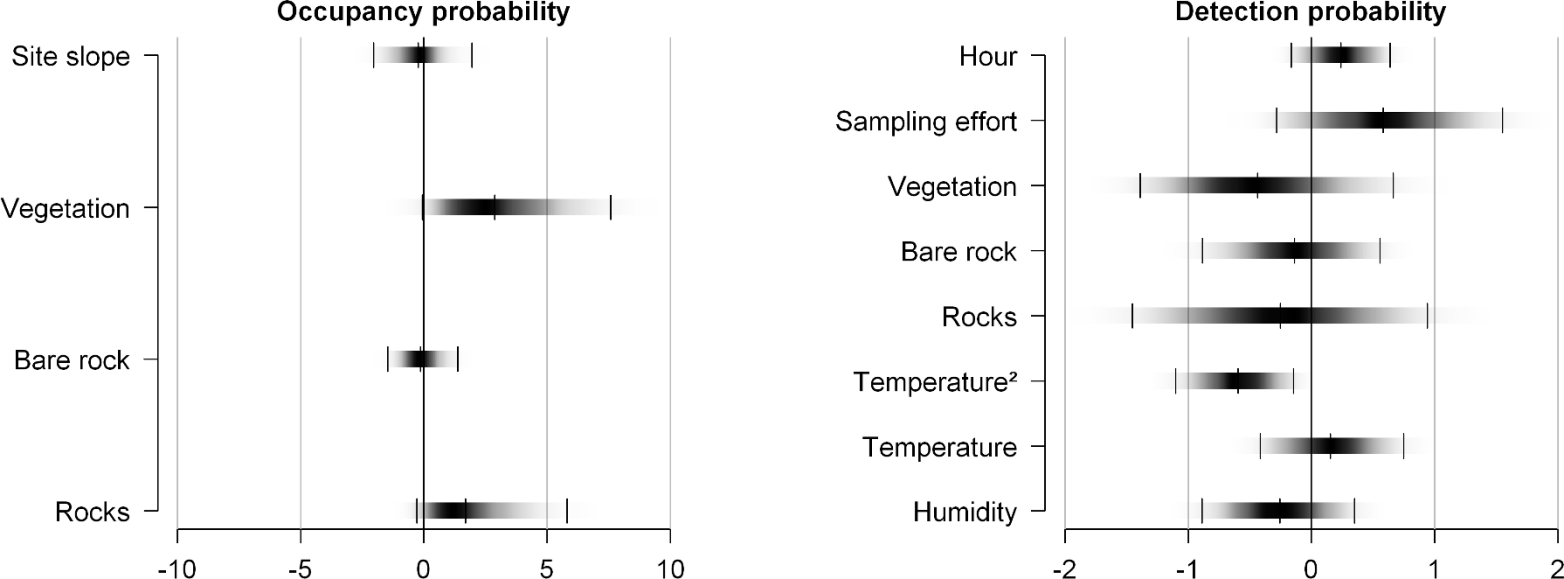
Posterior density of the coefficients for occupancy and detectability. All regression coefficients are given on the *logit* scale. "Significance" is accepted when 95% CRI (shown by two thin vertical lines of each density plot) does not overlap 0. Note the different scales on x-axis for occupancy and detection.

When accounting for imperfect detection in the model, occupancy was estimated at a much higher value, 0.80 (CRI 0.58 to 0.95), with 77 sites (CRI 56 to 92) estimated as occupied by the red-belly toad, rather than the 38 observed. Most of the posterior mass of the coefficient of vegetation density was positive suggesting a positive effect of this covariate on occupancy probability although the CRI barely included zero (β_*vegetation*_ = 3.14, CRI -0.06 to 7.61, Fig 2; S4 Fig). Similarly, the number of loose rocks appeared to have a positive effect on occupancy, although the CRI again barely overlapped zero (β_*rocks*_ = 2.00, CRI -0.28 to 5.81, Fig 2; S4 Fig). The other covariates, bare rock surface (β_*barerock*_ = -0.11, CRI -1.46 to 1.37) and site slope (β_*slope*_ = -0.22, CRI -2.03 to 1.95) presented weak effects with their posterior distribution masses sitting right about on zero (Fig 2; S4 Fig).

In our SDM without imperfect detection, the number of loose rocks positively correlated to toad presence (β_*rocks*_ = 2.00, CRI 0.43 to 3.66; Table 1), while effort (β_*effort*_ = -3.04, CRI -6.19 to -0.13; Table 1) and humidity (β_*humidity*_ = -2.90, CRI -6.00 to -0.06; Table 1) presented negative effects on toad presence. The effect of vegetation on toad presence was almost 10 times smaller when ignoring detection errors (Table 1).

**Table 1:** Coefficient means and standard deviation from the SDM without imperfect detection and the Occupancy model. The asterisk denotes important size effects.

SDM (no imperfect detection)			Occupancy model			
			Ecological submodel		Observational submodel	
	mean	sd	mean	sd	mean	sd
rocks	2.00*	0.83	2.00*	1.50	-0.25	0.60
bare rock	-0.26	0.30	-0.11	0.73	-0.14	0.36
vegetation	0.37	0.30	3.14*	1.90	-0.42	0.52
slope	0.21	0.27	-0.22	0.97	-	-
humidity	-2.89*	1.44	-	-	-0.26	0.31
temperature	-1.65	1.42	-	-	0.15	0.29
temperature^2^	-5.16	3.06	-	-	-0.60*	0.24
effort	-3.04*	1.53	-	-	0.60	0.47
hour	0.07	1.05	-	-	0.23	0.21
rain	1.01	1.43	-	-	0.11	0.18

### Detection patterns in the red-belly toad

**Fig 3:**
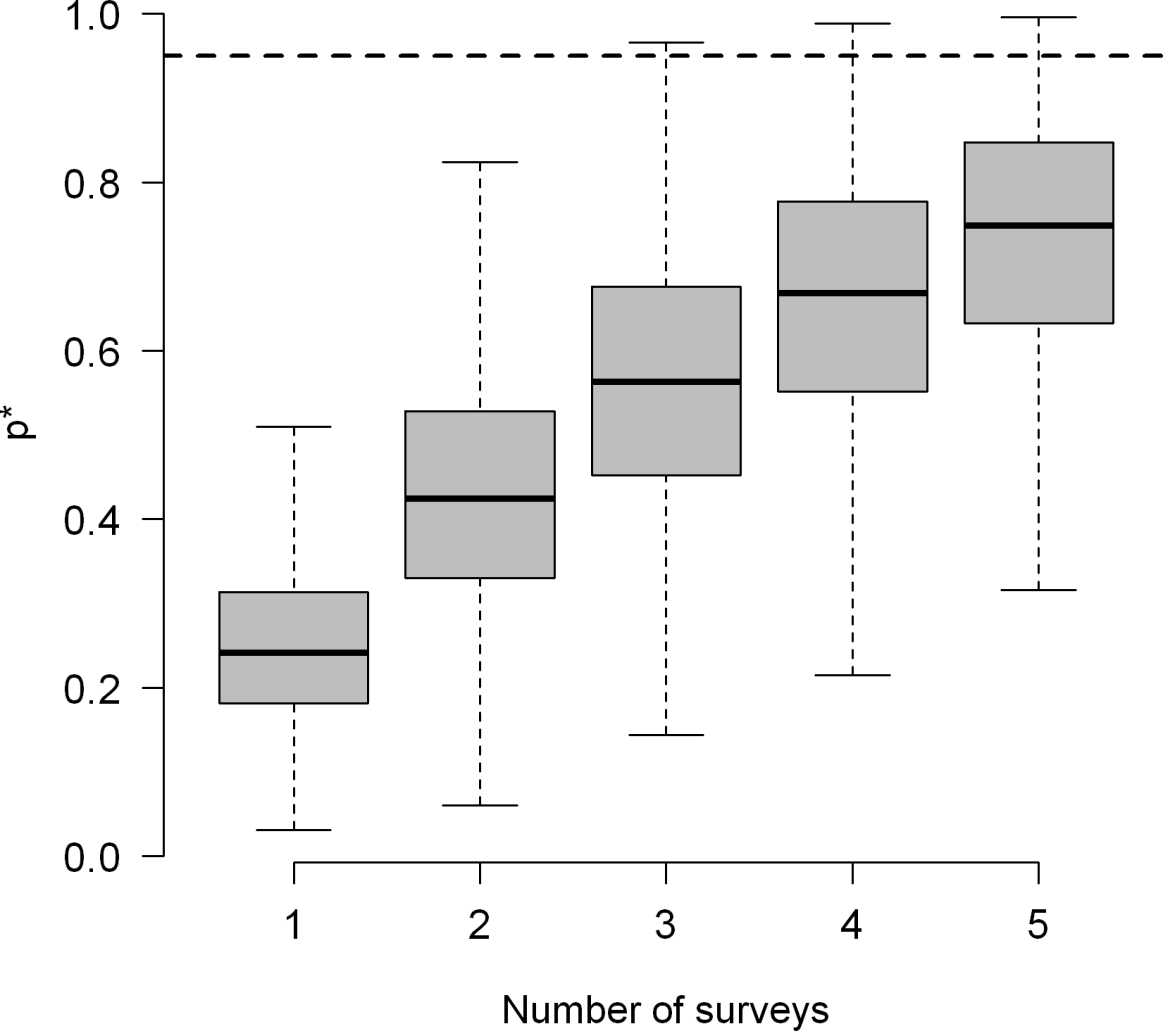
Probability to detecting *Melanophryniscus pachyrhynus* at a site at least once (p*) during *n* surveys. The dashed line indicates 95% certainty to detect the species when present. Boxes show the median value (center line in box), first and third quartiles (box ends). The 10th and 90th percentiles (represented by whiskers) and extreme values are also shown.

Mean detection probability (*p*) was estimated at 0.26 (CRI 0.10 to 0.50). According to our estimates, we would be able to detect the species at least once with three surveys on average (Fig 3), i.e., after three surveys, the combined probability of detection p* was greater than 0.95. Of the nine parameters included in the observational submodel for detection probability, only the quadratic effect of temperature (α_*temperature*^2^_ = -0.60, CRI -1.10 to -0.15) was important in our surveys (Fig 2; S5 Fig). Contrarily to our SDM without imperfect detection, the number of loose rocks and vegetation had a negative effect on detection probability, while effort had a positive effect (Table 1; Fig 2). The standard deviation of the posterior distribution mass of the random effects (*sd*.*alpha0*) was 1.68 (CRI 0.62 to 2.74) showing substantial heterogeneity on occupancy by the red-belly toad among sites.

## Discussion

Here, we used an occupancy modeling approach at a fine-scale to understand how the red-belly toad is distributed on a typical area of rocky outcrops. Fine-scale studies provide important information for species distribution because they generate knowledge on resource use and habitat management. Our study is the first evaluating the effects of microhabitat use while explicitly accounting for observational errors in this species, and we were able to shed light on local ecological requirements.

Accounting for non-detection sampling errors has been strongly recommended for field studies [26, 48, 54] and our estimates benefited from that, given the detection-corrected estimate of occupancy was twice as large as the naive occupancy estimate that ignored detection (0.39) (0.80, CRI 0.58 to 0.96). Occupancy estimates were much higher than expected, but this was due to the secretive habits of the species outside of the breeding season [29]. Two out of three covariates, vegetation density and number of loose rocks, were fitted in both occupancy and detection parameters, and presented opposite results, although their effect sizes differed (and strictly, were not "significant", since there 95% CRIs contained zero). Interestingly, higher values of both tended to increase occupancy probability but decreased detection probability. This highlights the importance of explicitly accounting for detection errors, otherwise the effect of both covariates would be underestimated.

### Habitat relationships in the red-belly toad

We considered only open areas containing rocky outcrops because we were interested in habitat use at a fine scale. We found that vegetation density had the most important effect on species habitat occupancy. The vegetation may play an important role on different aspects of the biology of the red-belly toad. First, it works as a protective barrier against the wind [55] maintaining air humidity high, which is especially important in harsh environments. Amphibians are often restricted to habitats with relative high humidity due to the need to control water balance [56]. Dense vegetation in open areas acts indirectly to control microclimatic variations by increasing relative humidity by up to 50% [57] and attenuating temperatures [58]. Second, in open areas dense vegetation may also provide both food resources and protection from aerial predators [59–61]. Ants, one of the main prey of the red-belly toads [62], respond positively to vegetation [63]. Third, vegetation cover surrounding breeding sites may generate a more stable thermocline for migration movements, also increasing tadpole survival by reducing desiccation risk [21, 64].

As a dominant feature in our study area, we included the rock element in our analysis in the form of two different covariates. Both the bare rock surface and the number of loose rocks on sites showed ‘non-significant’, but opposite, effects on occupancy probability. The size effect of bare rock surface on occupancy was small and negative, whereas the number of loose rocks had a more important size and positive effect. The bare rock surfaces within sites may be challenging environments for amphibians, reflecting generally poor conditions (e.g., high temperatures, low soil moisture, limited vegetation coverage) as shown for salamanders before [43]. On the other hand, loose rocks may be key for individuals seeking protection, food and regulating its physiological needs [36–37, 61]. Amphibians from rocky environments are often related to moist microhabitats, such as depressions, cracks and streams over rocks, vegetation, permanent steps and shaded pools [37]. Thus, heterogeneity seems to play an important role on amphibian occupancy on rocky outcrops, something that can only be revealed by fine-scale studies.

### Detection patterns in the red-belly toad

We had expected opposite effects of air humidity and temperature on detection probability since amphibians in general are often related to humid microhabitats with mild temperatures [65–66]. We also included the vegetation density, the bare rock surface, the number of loose rocks per site, sampling effort and time as potential covariates influencing detection probability. The quadratic effect of temperature was the most important among the covariates.

The relationship between detection probability and air temperature was not linear and peaked at around 11 °C (S5 Fig). Air temperature may be among the most important factors posing challenges for amphibians by determining activity periods, especially in high-elevation sites [67–68]. Breeding activity was also associated with air temperature in other red-belly toads [64, 68–69], such as *Melanophryniscus aff*. *montevidensis* whose detection was lower on breeding sites with high temperatures [70]. A bimodal activity pattern may represent an adaptation of the genus *Melanophryniscus* for extreme environments characterized by high thermal amplitude since avoidance of extremes air temperatures and sun radiation during the middle of the day seems to be common on these species [19].

Amphibians present different strategies to effectively reduce exposure to harsh environmental conditions [68]. Microhabitat features in open areas, such as vegetation structure, may be essential for toad survival and activity. Vegetation density had a negative, although weak, effect on detectability, suggesting that habitat complexity could impose difficulty to find individuals. The association between vegetation and the loose rocks seems to be important since toad species reduce activity in high temperatures, retreating into the vegetation to maintaining water balance [71]. Vertical movement is known for different groups, including salamanders, who migrate into underground retreats on rocky outcrop environments because of high temperatures [72–74]. Vertical movement is also described for anurans [75] and such temporary unavailability together with habitat complexity may lead to the conclusion of elusiveness or even rarity, but this may be misleading. The red-belly toads are considered elusive species, and vertical movemement may be a candidate hypothesys to explain such elusiveness given our results, including the finding of a captivity individual found buried at 20 cm below ground (KG pers. obs). Extreme temperatures, both low and high, may lead individuals to hide for long periods.

### Concluding remarks

Our occupancy model is an important step in better understanding the local distribution of the little known South American red-belly toad from the rocky outcrops of the Pampa biome. Our study included a rigorous spatial sampling protocol along with replicated surveys to most sample units, enabling us to adopt occupancy modeling to study habitat relationships free from distorting effects of imperfect detection. Therefore, our study may be useful as a functional protocol for surveys of this and similar species, and provided the first quantitative descriptions of habitat selection for the red-belly toad. We acknowledge the relevance of occupancy modeling framework for studies from a detailed perspective of spatial patterns and believe these results may provide managers information for enhancing the conservation of the red-belly toad and its associated habitats.

Predictive habitat selection models are valuable because management activities often manipulate habitat [76–77]. Also, understanding fine-scale habitat use is essential for the connection between species distribution and local resource availability [78]. Fine-scale effects are especially relevant for complex landscapes where habitat varies widely in quality, quantity and configuration [79]. Variation in habitat quality should influence species distribution across scales because high-quality habitats confer higher number of patches available for occupancy [13]. However, different process and mechanisms are likely to explain the patterns in biological systems [7] and thus, no description of the predictability of the environment makes sense without referencing the range of scales and the organisms involved [80].

Habitat structure is affected by a combination of natural and man-induced factors that may interact over multiple spatiotemporal scales affecting species distribution [81]. For instance, burning and livestock are common practices in the South American Pampa biome and cattle grazing has been suggested as a way to preserve the natural characteristics of the grasslands, controlling shrub vegetation growth [82–83]. On the other hand, cattle exclusion may positively affects amphibian populations by allowing vegetation growth [84–85]. By studying local populations and fine-scale habitat use, we are able to provide important information to predict population preferences, which in turn may help comprehending potential local declines and guiding effective conservation strategies.

## Supporting information

S1 Fig General view of the study area, including occasional slopes with hills and rock outcrops surrounded by ephemeral ponds, grasslands, shrub vegetation and gallery forests. The valleys of the hills consist of native forest and, in smaller proportions, recently eucalypt plantation woodlands.
(TIF)

S2 Fig Vegetation density and bare rocky surface data collection. Twelve repeated samples (grey squares) were systematically obtained inside each site, according to site slope and it´s center (C), as shown. Each sample was divided into 16-unit sub-squares scattered. We counted the number of sub-squares touching shrub plants at 50cm high and the number that corresponded to bare rocky surface. Finally, we calculated an index of both covariates (vegetation density and bare rock) based on the proportion of total sub-squares with these habitat features.
(TIF)

S3 Fig JAGS code. (PDF)

S4 Fig Predictions of occupancy probability in relation to the covariates in the model for the red-belly toad. Black lines represent the mean prediction and gray lines are 200 random draws from the posterior distribution of the predictions as a way to depict the prediction uncertainty.
(TIFF)

S5 Fig Predictions of detection probability in relation to the covariates in the model for the red-belly toad. Black lines represent the mean prediction and gray lines are 200 random draws from the posterior distribution of the predictions as a way to depict the prediction uncertainty.
(TIFF)
