# Supplementary figures and images for: Unraveling fine-scale habitat use for secretive species: When and where toads are found when not breeding
Source: PLoS One. 2018 Oct 8;13(10):e0205304. doi: 10.1371/journal.pone.0205304 (PMC6175507; doi:10.1371/journal.pone.0205304)

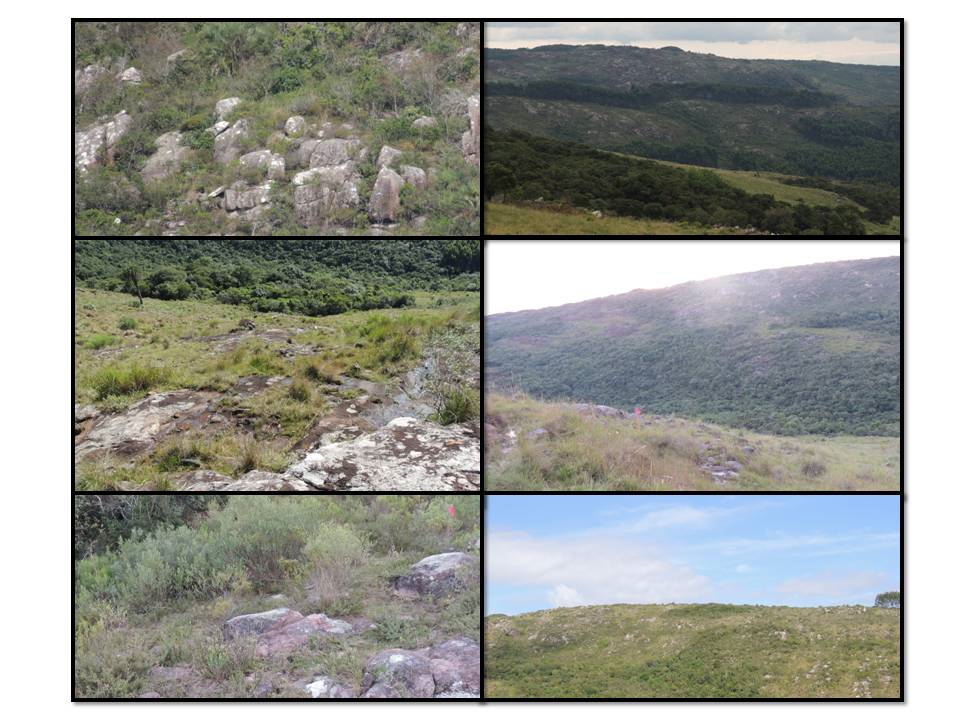

Supplement: S1 Fig — The valleys of the hills consist of native forest and, in smaller proportions, recently eucalypt plantation woodlands. (TIF) [file pone.0205304.s001.tif]

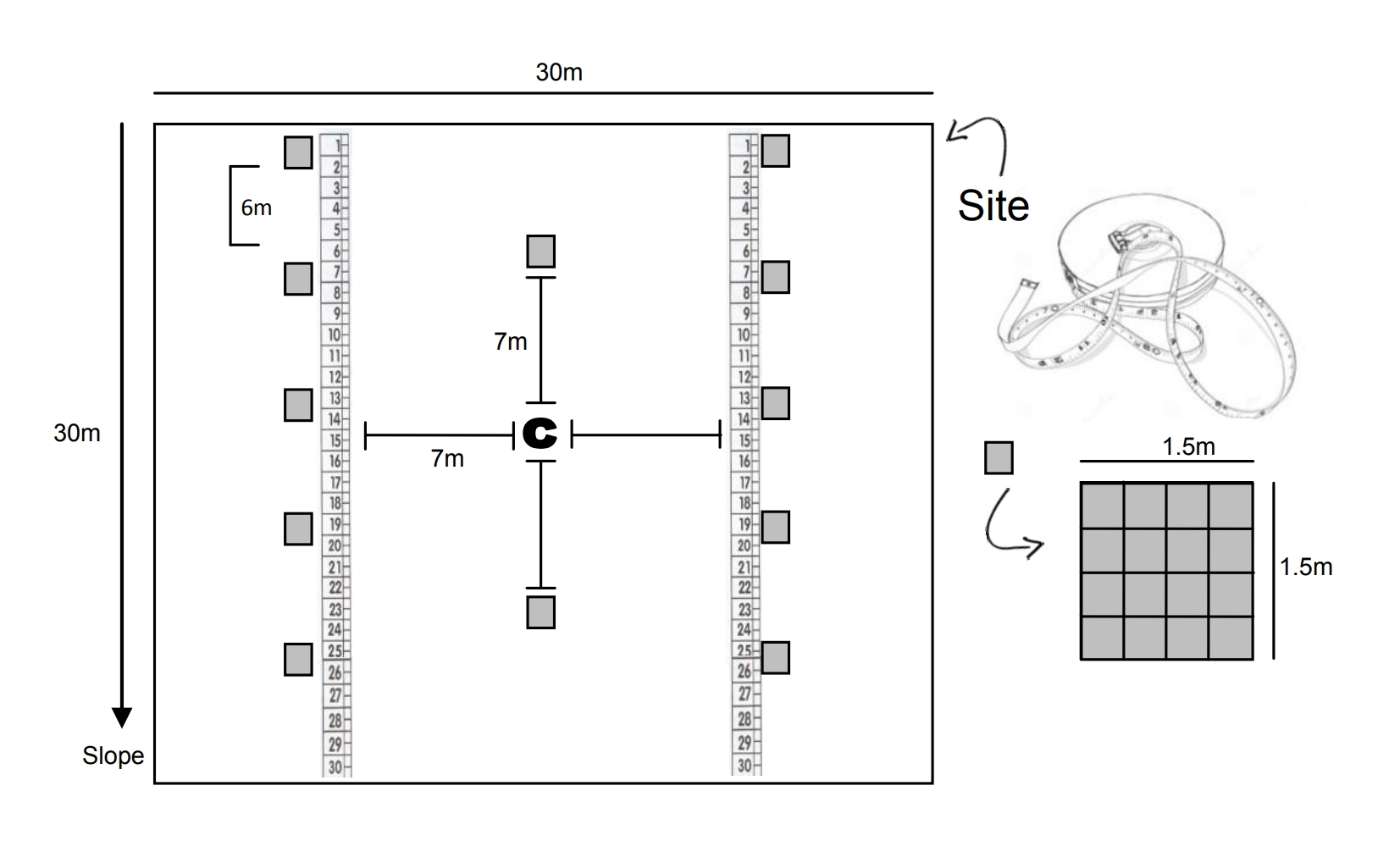

Supplement: S2 Fig — Twelve repeated samples (grey squares) were systematically obtained inside each site, according to site slope and it´s center (C), as shown. Each sample was divided into 16-unit sub-squares scattered. We counted the number of sub-squares touching shrub plants at 50cm high and the number that corresponded to bare rocky surface. Finally, we calculated an index of both covariates (vegetation density and bare rock) based on the proportion of total sub-squares with these habitat features. (TIF) [file pone.0205304.s002.tif]

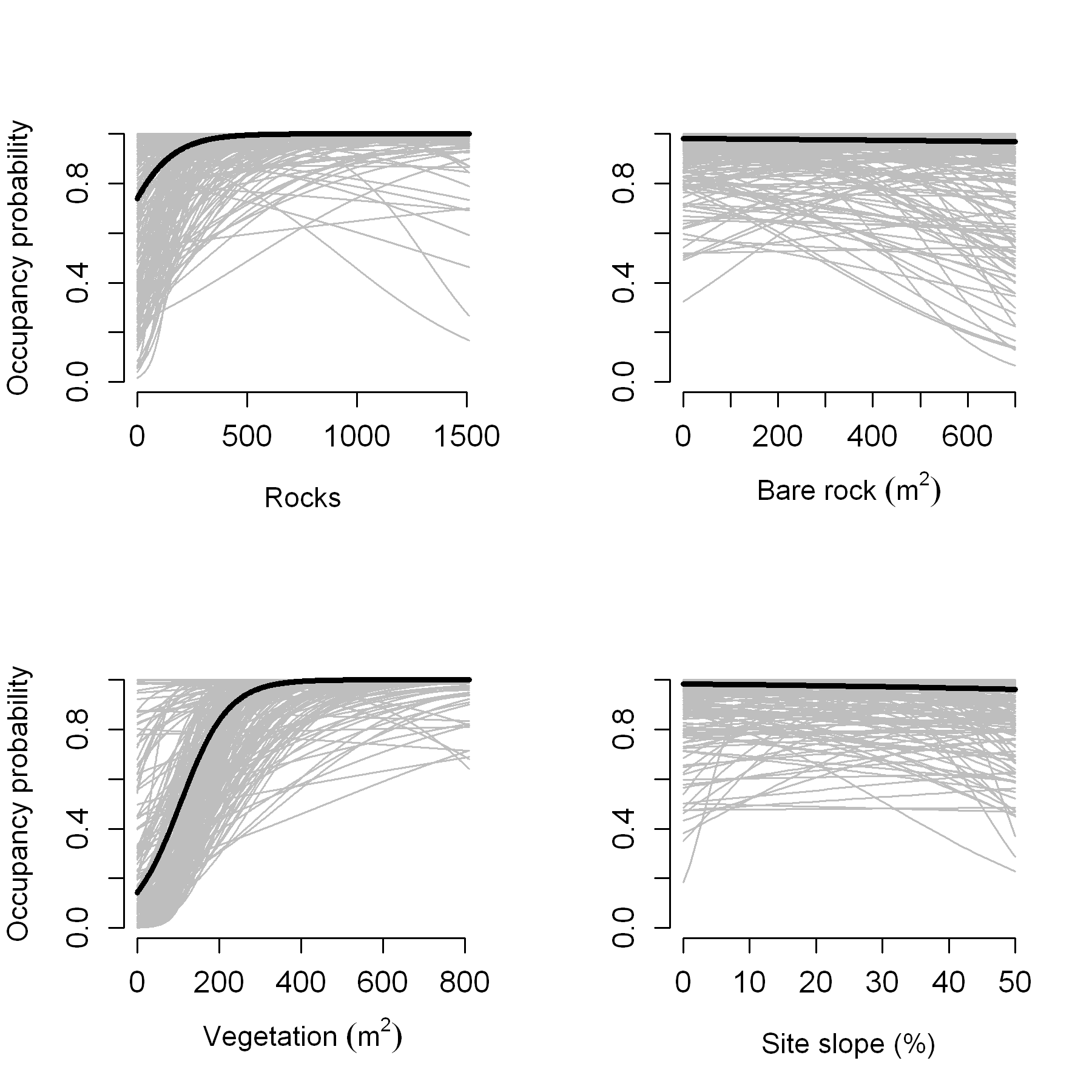

Supplement: S4 Fig — Black lines represent the mean prediction and gray lines are 200 random draws from the posterior distribution of the predictions as a way to depict the prediction uncertainty. (TIFF) [file pone.0205304.s004.tiff]

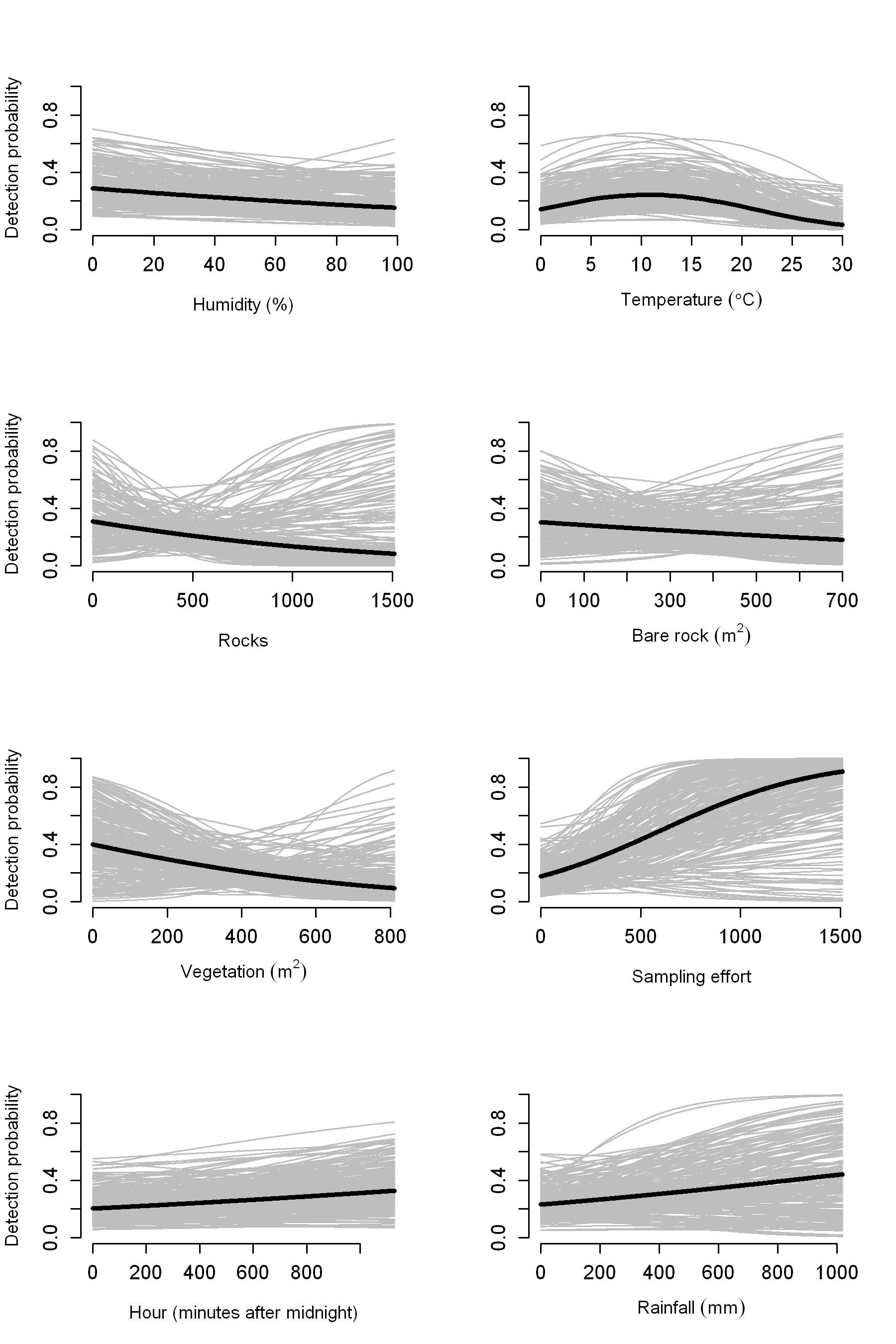

Supplement: S5 Fig — Black lines represent the mean prediction and gray lines are 200 random draws from the posterior distribution of the predictions as a way to depict the prediction uncertainty. (TIFF) [file pone.0205304.s005.tiff]
