# Supplementary material for: Unraveling fine-scale habitat use for secretive species: When and where toads are found when not breeding
Source: PLoS One. 2018 Oct 8;13(10):e0205304. doi: 10.1371/journal.pone.0205304 (PMC6175507; doi:10.1371/journal.pone.0205304)
Supplement: S3 Fig — (PDF) [file pone.0205304.s003.pdf]

```
# Write a text file in R with BUGS code of model
sink("model2.txt")
cat("
    model {

  # Priors
    #Occupancy
    mean.psi ~ dunif(0, 1) #occupancy intercept on prob. scale
    beta0 <- logit(mean.psi)#occupancy intercept
    beta1 ~ dunif(-10,10)  #occupancy slope on rocks
    beta2 ~ dunif(-10,10)  #occupancy slope on barerock
    beta3 ~ dunif(-10,10)  #occupancy slope on vegetation
    beta4 ~ dunif(-10,10)  #occupancy slope on siteslope


    #Detection
    mean.p ~ dunif(0, 1)    #Detection intercept on prob. scale
    alpha0 <- logit(mean.p) #Detection intercept
    alpha1 ~ dunif(-10,10)  #Detection slope on humidity
    alpha2 ~ dunif(-10,10)  #Detection slope on temperature
    alpha3 ~ dunif(-10,10)  #detection slope on temperature (^2)
    alpha4 ~ dunif(-10,10)  #detection slope on rocks
    alpha5 ~ dunif(-10,10)  #detection slope on barerock
    alpha6 ~ dunif(-10,10)  #detection slope on vegetation
    alpha7 ~ dunif(-10,10)  #detection slope on samplingeffort
    alpha8 ~ dunif(-10,10)  #detection slope on hour
    alpha9 ~ dunif(-10,10)  #detection slope on rainfall

    tau.alpha0 <- 1 / (sd.alpha0 * sd.alpha0)  # sd hyperparam
    sd.alpha0 ~ dunif(0,10)


  # Likelihood

    # Loop over sites
    for (i in 1:nsite) {
    z[i] ~ dbern(psi[i])# state model
    logit(psi[i]) <- beta0 + beta1 * rocks[i] +
    beta2 * barerock[i] + beta3 * vegetation[i] +
    beta4 * siteslope[i]
    eps.p[i] ~ dnorm(0,tau.alpha0)

    # Loop over replicate surveys
    for (j in 1:nrep) {
    CH[i,j] ~ dbern(z[i] * p[i,j])   # Observation model
    logit(p[i,j]) <- alpha0 + alpha1 * humidity[i,j] +
    alpha2 * temperature[i,j] + alpha3 * pow(temperature[i,j],2)
    + alpha4 * rocks[i] + alpha5 * barerock[i] +
    alpha6 * vegetation[i] + alpha7 * samplingeffort[i,j] +
    alpha8 * hour[i,j] + alpha9 * rainfall[i,j] + eps.p[i]
    }
    }

  # Derived quantities
    N.occ <- sum(z[]) # Number of occupied sites
    psi.mean <- N.occ/nsite # Proportion of occupied sites
    p.mean <- exp (alpha0) / (1 + exp (alpha0)) # mean detection

    }

    ",fill = TRUE)
```

```
62
63  sink()
64
65  # Bundle data and summarize
66  str(win.data <- list(CH = CH, nsite = nrow(CH), nrep = ncol(CH),
67  rocks=rocks, barerock=barerock, vegetation=vegetation,
68  siteslope=siteslope,humidity=humidity, temperature=temperature,
69  hour=hour, rainfall=rainfall, samplingeffort=samplingeffort))
70
71  # Initial values
72  zst <- apply(CH, 1, max, na.rm=TRUE)# Avoid model/inits conflict
73  inits <- function(){list(z = zst, mean.psi = runif(1), beta1 =
74  runif(1), beta2 = runif(1), beta3 = runif(1), beta4 = runif(1),
75  mean.p = runif(1), alpha1 = runif(1), alpha2 = runif(1), alpha3 =
76  runif(1), alpha4 = runif(1), alpha5 = runif(1),
77  alpha6 = runif(1), alpha7 = runif(1), alpha8 = runif(1), alpha9 =
78  runif(1),  sd.alpha0 = runif(1,0,10))}
79
80
81  # Parameters monitored
82  params <- c("alpha0","alpha1", "alpha2", "alpha3", "alpha4", "alpha5",
83  "alpha6", "alpha7", "alpha8","alpha9",
84  "beta0","beta1", "beta2", "beta3", "beta4",
85  "N.occ", "psi.mean", "p.mean",
86  "sd.alpha0")
87
88
89  # MCMC settings
90  ni <- 200000   ;   nt <- 50   ;   nb <- 100000   ;   nc <-3
91
92  #Call JAGS from R
93  library("jagsUI")
94  m2 <- jags(win.data, inits, params, "model2.txt", n.chains = nc,n.thin
95  = nt, n.iter = ni, n.burnin = nb)
```
